# Transparent and flexible capacitors based on nanolaminate Al_2_O_3_/TiO_2_/Al_2_O_3_

**DOI:** 10.1186/s11671-015-0784-8

**Published:** 2015-02-18

**Authors:** Guozhen Zhang, Hao Wu, Chao Chen, Ti Wang, Jin Yue, Chang Liu

**Affiliations:** Key Laboratory of Artificial Micro- and Nano-structures of Ministry of Education, School of Physics and Technology, Wuhan University, Wuhan, 430072 People’s Republic of China

**Keywords:** Transparent capacitors, Atomic layer deposition, Flexible devices

## Abstract

Transparent and flexible capacitors based on nanolaminate Al_2_O_3_/TiO_2_/Al_2_O_3_ dielectrics have been fabricated on indium tin oxide-coated polyethylene naphthalate substrates by atomic layer deposition. A capacitance density of 7.8 fF/μm^2^ at 10 KHz was obtained, corresponding to a dielectric constant of 26.3. Moreover, a low leakage current density of 3.9 × 10^−8^ A/cm^2^ at 1 V has been realized. Bending test shows that the capacitors have better performances in concave conditions than in convex conditions. The capacitors exhibit an average optical transmittance of about 70% in visible range and thus open the door for applications in transparent and flexible integrated circuits.

## Background

In recent years, there has been considerable interest in transparent electronic devices formed on plastic or other bendable substrates to meet the growing demand for low-cost, large-scale, high-flexibility, and lightweight devices [1]. Examples of transparent and flexible applications include flat-panel displays, e-papers, solar cells, and wearable computers [2,3]. In driven circuits of these transparent devices, capacitors play an important role such as storage capacitors in solar cell modules [4]. High-frequency charge–discharge capacitors in active matrix displays [5,6], decoupling capacitors for microprocessors, filter and analog capacitors working with other electronic components to realize various logical functions.

High-*k* oxides are promising candidates for materials used in transparent electronics due to their excellent properties such as large dielectric constants, high optical transmittance, and simple preparation methods [7]. Capacitors with large capacitance, reduced feature size, and low power consumption can be realized by using high-*k* materials as dielectrics [8,9]. Many high-*k* materials, including Al_2_O_3_ [10], HfO_2_ [11], TiO_2_ [12], and various hybrid dielectric stacks [13], have been widely investigated. Among them, TiO_2_ is an attractive material due to its transparency and a large dielectric constant of about 180 in rutile phase. However, its leakage current is also very large because of its relatively small bandgap and n-type semiconductor nature. Many approaches have been made to reduce the leakage current. Among them, the sandwich structure of nanolaminate Al_2_O_3_/TiO_2_/Al_2_O_3_ (ATA) [14] has been commonly used because Al_2_O_3_ has a large bandgap of about 8.9 eV, and its excellent passivation properties usually lead to high-quality interfaces. In our previous work, we demonstrated a kind of transparent capacitor with nanolaminate ATA as dielectrics and Al-doped ZnO (AZO) as electrodes on quartz glass [15]. A maximal capacitance density of 14 fF/μm^2^ at 1 KHz was obtained. Moreover, the leakage current density at 1 V was reduced to an ultralow value of 2.1 × 10^−9^ A/cm^2^.

Many techniques have been used to deposit dielectric thin films, such as chemical vapor deposition (CVD) [16], pulsed laser deposition (PLD) [17], magnetron sputtering [18], and sol–gel spin coating method [19]. However, CVD methods usually require a high growth temperature which is not suitable for many flexible substrates. For magnetron sputtering and PLD methods, the quality of films is usually proportional to the deposition temperature. In addition, physical damages on the film may be caused by high-energy particles in sputtering or PLD process. In 2009, Meena et al. used a sol–gel spin-coating process to deposit HfO_2_ films on a Cr/Au-coated flexible polymide substrate at room temperature [19]. This capacitor device exhibited excellent electrical properties under various bending conditions. However, this method is not suitable for fabricating very thin dielectric films which are continuous and have no pinhole because it is difficult to accurately control the sol–gel process. In addition, the Cr/Au electrodes limit their applications in transparent electronics. Atomic layer deposition (ALD) is an alternative way to deposit high-quality dielectric films [20]. It employs an intrinsic self-limiting growth mode to deposit thin films with atomic layer accuracy and demonstrates many advantages such as accurate thickness control, high uniformity over a large area, low defect density, and good reproducibility. In addition, the low growth temperature and large-size chamber make ALD a very efficient way to deposit large-area films on flexible substrates, which is very beneficial for mass production.

In this work, the entire structures of capacitors were *in situ* grown on an indium tin oxide (ITO)-coated polyethylene naphthalate (PEN) substrate by ALD. Here, the nanolaminate ATA films were used as the dielectric layer, and AZO films were selected as the top electrode. A capacitance density of 7.8 fF/μm^2^ at 10 KHz was obtained. In addition, the capacitor device shows a low leakage current density of 3.9 × 10^−8^ A/cm^2^ at 1 V. A bending test was conducted to examine the flexibility. The leakage mechanism was also investigated.

## Methods

The ITO-coated PEN substrate was purchased from HeptaChroma (Dalian, China). The sheet resistance of ITO is about 15 Ω, which is low enough to serve as the bottom electrode. Firstly, the ITO/PEN substrate was cleaned ultrasonically in heated ethanol (60°C) for 30 min followed by deionized water rinse to remove the surface contaminants. Then high-pressure N_2_ gas was used to blow off the water and any remaining particles from the ITO surface. After that, a small part of ITO films was protected by the Kapton tape to serve as the probe position during subsequent electrical measurements. Al_2_O_3_/TiO_2_/Al_2_O_3_ films (5/20/5 nm) were then deposited on the ITO surface by ALD. Here, the 5 and 20 nm represent the thicknesses of Al_2_O_3_ and TiO_2_ films, respectively. Al_2_O_3_ films were grown at 150°C by using the precursors of trimethyl aluminum (TMA) and H_2_O. The growth rate was about 0.07 nm/cycle and 72 cycles were used for the thickness of 5 nm. Tetrakis-dimethylamido titanium (TDMAT) and H_2_O were used to grow TiO_2_ at 125°C. The growth rate was about 0.05 nm/cycle, and the films were grown with 400 cycles. After that, AZO films with a thickness of about 200 nm were deposited at 150°C as the top electrode. The AZO films were composed of 50 periods. Each period included 20 cycles of ZnO and 1 cycle of Al_2_O_3_. Diethyl zinc (DEZn) and deionized water were used to deposit ZnO films with a growth rate of 0.2 nm/cycle. The growing conditions of Al_2_O_3_ in AZO films were the same as that of Al_2_O_3_ in ATA films. A spectroscopic ellipsometer (J. A. Woollam alpha-SE, J. A. Woollam Co. Inc., Lincoln, NE, USA) was used to determine the film thicknesses. Standard photolithography and wet etching process were used to define the capacitor areas. The final capacitor device was approximately 100 × 100 μm^2^ in area. The capacitance density versus voltage (*C*-*V*) and leakage current density versus voltage (*I*-*V*) characteristics were measured by a semiconductor device analyzer (Keithley 4200, Keithley Instruments, Solon, OH, USA). The optical transmittance was measured in a wavelength range of 300 to 800 nm by using a UV–VIS-NIR spectrophotometer (Varian Cary 5000, Triad Scientific, Manasquan, NJ, USA). The surface morphology of ITO and ATA films was measured by an atomic force microscopy (SPM-9500 J3, Shimadzu, Kyoto, Japan).

## Results and discussion

**Figure 1:**
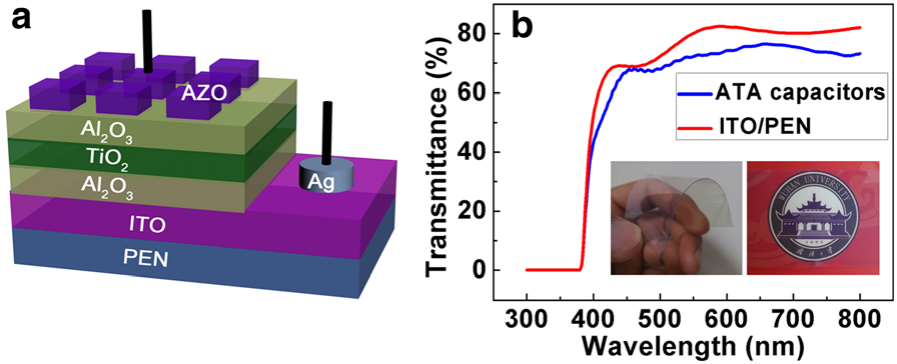
**Schematic diagram of the flexible capacitors and transmittance spectra in the wavelength range from 300 to 800 nm. (a)** The schematic diagram of transparent and flexible capacitors. **(b)** The optical transmittance spectra of the capacitor device. The inset of **(b)** is the photograph of the capacitor device.

Figure 1a shows the schematic diagram of the flexible capacitors. Figure 1b shows the transmittance spectra in the wavelength range from 300 to 800 nm. An average optical transmittance of more than 70% was observed, which is valuable for applications in transparent electronics. As shown from the inset of Figure 1b, the Chinese characters “Wuhan University” can be clearly seen through the transparent capacitor device.

**Figure 2:**
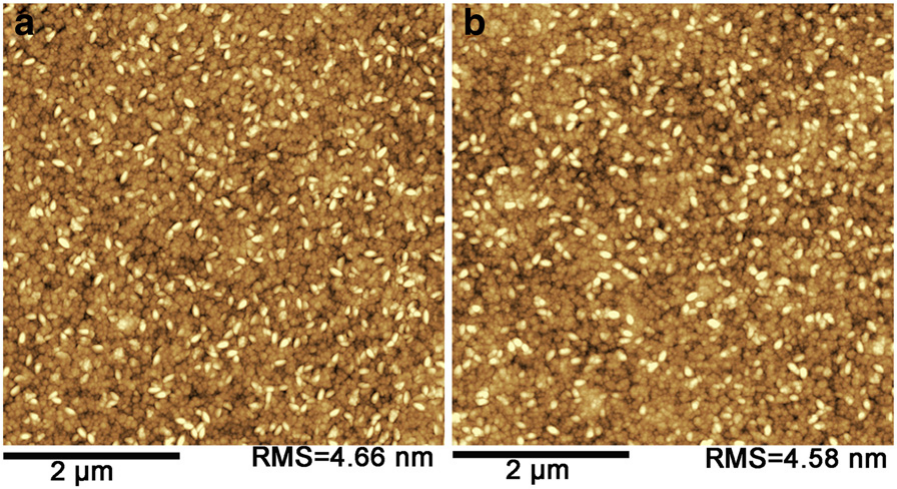
**AFM images of the ITO/PEN surface (a) and ATA dielectrics surface (b).**

Figure 2a shows the 5 × 5 μm^2^ atomic force microcopy images of the ITO/PEN surface. The root mean square (RMS) value of the surface roughness was about 4.7 nm. The relatively big surface roughness resulted from the high-density ITO crystalline grains which are small white particles shown in Figure 2a. After deposition of ATA thin films, the RMS value was slightly reduced to 4.6 nm and the small crystalline grains were increased slightly in size, as shown in Figure 2b. This suggests that uniform coatings of ATA films have been realized on the ITO bottom electrode.

**Figure 3:**
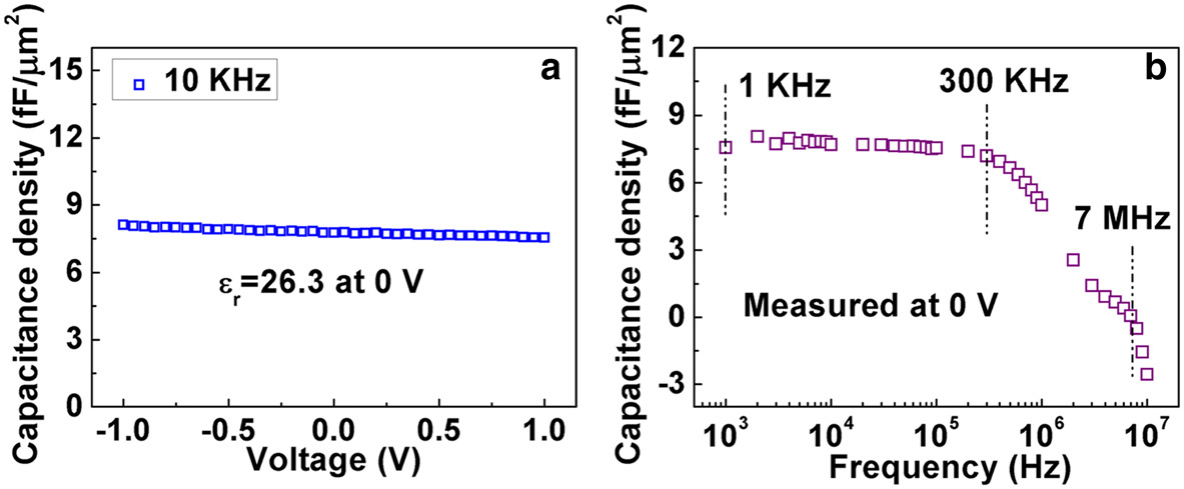
***C*** **-** ***V*** **and** ***C*** **-** ***F*** **characteristics of the capacitor device at frequencies from 1 KHz to 10 MHz. (a)** *C*-*V* characteristics of the capacitor device at 10 KHz. **(b)** *C*-*F* characteristics of the capacitor device from 1 KHz to 10 MHz.

**Figure 4:**
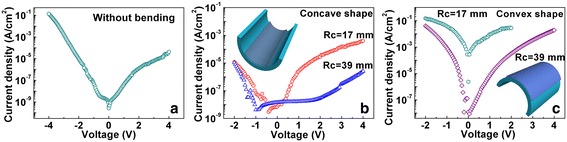
**I-V characteristics and bending test of the capacitor device. (a)** *I*-*V* characteristics of the capacitor device without bending from −4 to 4 V and bending test of the capacitor device in concave **(b)** and convex **(c)** conditions.

Figure 3a shows the *C*-*V* characteristics of ATA capacitors measured at 10 KHz. The capacitance density reached 7.8 fF/μm^2^ at 0 V, corresponding to a dielectric constant of 26.3. Figure 3b demonstrates the *C*-*F* characteristics of the capacitor device at frequencies from 1 KHz to 10 MHz when the direct voltage was fixed at 0 V. The capacitance density keeps almost constant from 1 to 300 KHz. The little dielectric loss at low frequencies can be attributed to the relatively low leakage current density which is 3.9 × 10^−8^ A/cm^2^ at 1 V, as shown in Figure 4a. However, the capacitance density dropped significantly when the frequency was above 300 KHz. The dielectric loss happened at high frequencies mainly because of the relatively high sheet resistance of AZO films (about 70 Ω). Hence, the flexible capacitor device can work steadily at frequencies between 1 and 300 KHz.

**Figure 5:**
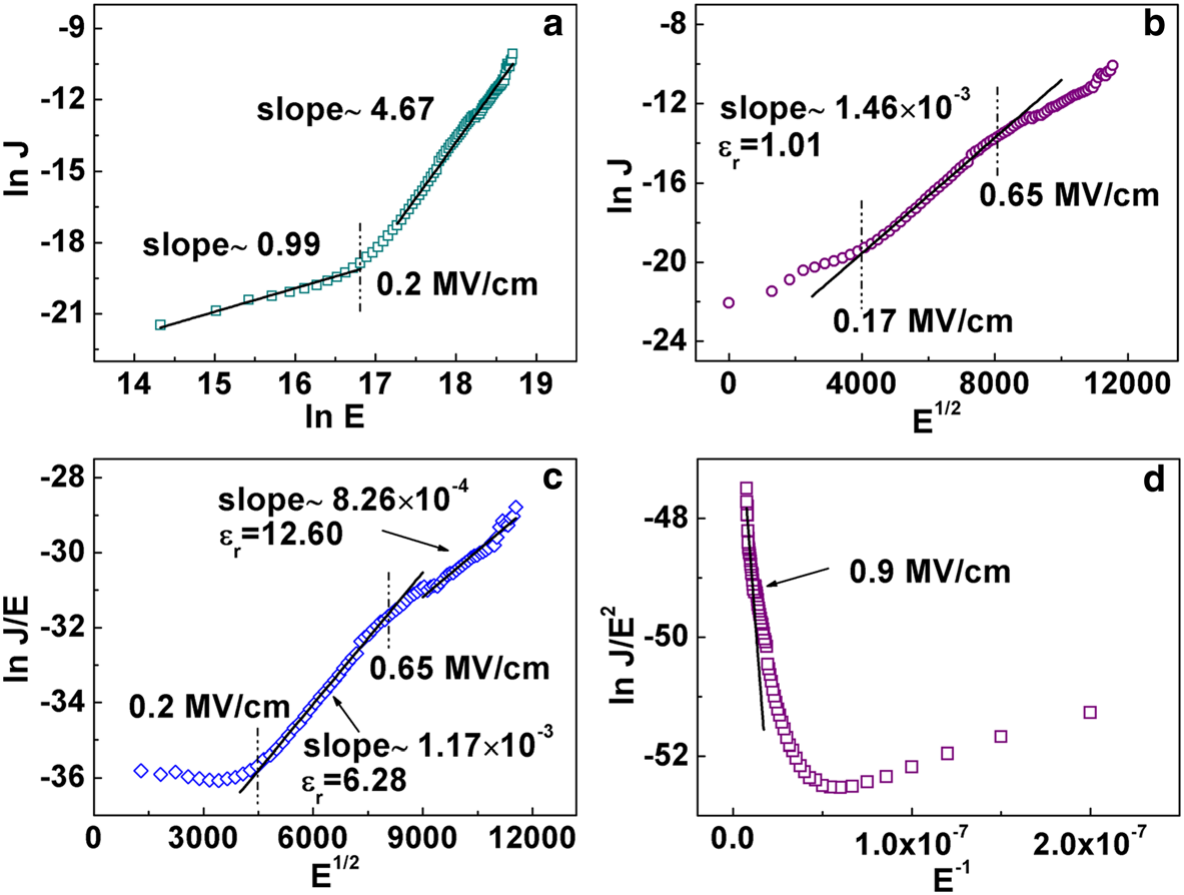
**The leakage current analysis of the capacitor device. (a)** Ohmic plots. **(b)** Schottky plots. **(c)** F-P plots. **(d)** F-N tunneling plots.

It is very meaningful to understand the leakage mechanisms which are useful in determining how to effectively control the leakage current. As shown in Figure 5a, the slope of ln(*J*) vs ln(*E*) is close to 1, which suggests that ohmic behavior is dominant when the field is below 0.2 MV/cm. When the field is above 0.2 MV/cm, the leakage conduction satisfies neither ohmic nor space-charge-limited-current (SCLC) mechanism. Schottky or Frenkel-Poole (F-P) emission models are commonly used to describe the leakage conduction at low or moderate electric fields [21]. The relationship between ln(*J*) and *E*^1/2^ should be linear if the leakage conduction is governed by Schottky emission. Similarly, the plot of ln(*J*/*E*) ~ *E*^1/2^ would present a linear relationship when the leakage conduction follows F-P emission. Figure 5b shows the Schottky plots with ln(*J*) ~ *E*^1/2^. A linear relationship is observed when the field is increased from 0.17 to 0.65 MV/cm. But the fitted relative dielectric constant is 1.01, which is not reasonable comparing with the measured one (26.3). Hence, the Schottky emission exists but is not the main leakage mechanism. Figure 5c shows the F-P plots with ln(*J*/*E*) ~ *E*^1/2^. The linear relationship suggests that F-P emission also exists in the field range from 0.17 to 0.65 MV/cm. However, the fitted dielectric constant still cannot match with the measured ones. Therefore, Schottky and F-P emissions coexist in the field range from 0.17 to 0.65 MV/cm, and neither of them governs the leakage current conduction. Fowler-Nordheim (F-N) tunneling mechanism is usually used to describe the leakage conduction in high field range. Figure 5d shows the F-N plots with ln(*J*/*E*^2^) ~ *E*^−1^. It is found that F-N tunneling happens when the electric field is above 0.9 MV/cm.

In order to verify the flexibility of ATA capacitors, bending test was conducted with both concave and convex conditions. Two radiuses of curvature (*R*_c_) of 17 and 39 mm were selected. In concave conditions, when the entire device was under the compressive stress transmitted from the ITO/PEN substrate, the leakage current remained almost unchanged when *R*_c_ was 39 mm, as shown in Figure 4b. In convex conditions, when the device structure was under the tensile stress from the ITO/PEN substrate, the leakage current density was bigger than that of concave conditions at the same *R*_c_. This is because ITO films are fragile and have a big thickness (about 300 nm), which leads to a large tensile stress at the interface between ITO and ATA films in convex conditions. However, in concave conditions, the compressive stress between ITO and ATA films was equal to the tensile stress transmitted from the top AZO electrodes, which was much smaller than the tensile stress transmitted from ITO/PEN substrates in convex conditions due to the dielectrics with a small thickness and scattered top electrodes (100 μm^2^ × 100 μm^2^ each). The tensile or compressive stress leads to many leakage pathways produced in ATA dielectric layer. When *R*_c_ was reduced to 17 mm, the leakage current became very large, as shown in Figure 4c. This is probably due to the formation of cracks in ITO films, which produced many local connections of top and bottom electrodes near the crack region and thus induce the failure of the capacitor device. Therefore, the ATA capacitors can be operated in limited bending conditions.

## Conclusions

In conclusion, we have successfully fabricated transparent ATA capacitors on flexible ITO/PEN substrate by ALD method. *C*-*V* measurements show a better capacitance density of 7.8 fF/fFm^2^ at 10 KHz, corresponding to a dielectric constant of 26.3. In addition, a low leakage current density of 3.9 × 10^−8^ A/cm^2^ at 1 V was obtained. By analyzing the leakage current, we conclude that ohmic behavior is the main mechanism in a low field range (*E* < 0.2 MV/cm), while Schottky and F-P emissions coexist in the field range from 0.17 to 0.65 MV/cm. F-N tunneling happens when the field is above 0.9 MV/cm. Bending test reveals that the flexible capacitors can be operated in limited bending conditions. The ATA transparent capacitors exhibit an average optical transmittance over 70% in the visible range, which opens the possibilities for applications in transparent and flexible integrated circuits.
